# Relationships among Body Condition, Insulin Resistance and Subcutaneous Adipose Tissue Gene Expression during the Grazing Season in Mares

**DOI:** 10.1371/journal.pone.0125968

**Published:** 2015-05-04

**Authors:** Shaimaa Selim, Kari Elo, Seija Jaakkola, Ninja Karikoski, Ray Boston, Tiina Reilas, Susanna Särkijärvi, Markku Saastamoinen, Tuomo Kokkonen

**Affiliations:** 1 Department of Agricultural Sciences, P.O. Box 28, FI-00014 University of Helsinki, Helsinki, Finland; 2 Department of Equine and Small Animal Medicine, P.O. Box 57, FI-00014 University of Helsinki, Helsinki, Finland; 3 Department of Clinical Studies, New Bolton Center, School of Veterinary Medicine, University of Pennsylvania, Philadelphia, PA 19104, United States of America; 4 Department of Green Technology, Natural Resources Institute Finland (Luke), Opistontie 10 A 1, FI-32100 Ypäjä, Finland; Wageningen UR Livestock Research, NETHERLANDS

## Abstract

Obesity and insulin resistance have been shown to be risk factors for laminitis in horses. The objective of the study was to determine the effect of changes in body condition during the grazing season on insulin resistance and the expression of genes associated with obesity and insulin resistance in subcutaneous adipose tissue (SAT). Sixteen Finnhorse mares were grazing either on cultivated high-yielding pasture (CG) or semi-natural grassland (NG) from the end of May to the beginning of September. Body measurements, intravenous glucose tolerance test (IVGTT), and neck and tailhead SAT gene expressions were measured in May and September. At the end of grazing, CG had higher median body condition score (7 vs. 5.4, interquartile range 0.25 vs. 0.43; *P=0*.*05*) and body weight (618 kg vs. 572 kg ± 10.21 (mean ± SEM); *P=0*.*02*), and larger waist circumference (*P=0*.*03*) than NG. Neck fat thickness was not different between treatments. However, tailhead fat thickness was smaller in CG compared to NG in May (*P=0*.*04*), but this difference disappeared in September. Greater basal and peak insulin concentrations, and faster glucose clearance rate (*P*=0.03) during IVGTT were observed in CG compared to NG in September. A greater decrease in plasma non-esterified fatty acids during IVGTT (*P<0*.*05*) was noticed in CG compared to NG after grazing. There was down-regulation of insulin receptor, retinol binding protein 4, leptin, and monocyte chemoattractant protein-1, and up-regulation of adiponectin (*ADIPOQ*), adiponectin receptor 1 and stearoyl-CoA desaturase (*SCD*) gene expressions in SAT of both groups during the grazing season (*P<0*.*05*). Positive correlations were observed between *ADIPOQ* and its receptors and between *SCD* and *ADIPOQ* in SAT (*P<0*.*01*). In conclusion, grazing on CG had a moderate effect on responses during IVGTT, but did not trigger insulin resistance. Significant temporal differences in gene expression profiles were observed during the grazing season.

## Introduction

Obesity is a common disease and welfare problem in equines [1]. The metabolic effects of obesity and its association with insulin resistance, laminitis, and increased inflammatory cytokine expression in equids are of increasing prevalence and importance. Equine metabolic syndrome is an endocrinopathic disease of horses and ponies characterized by obesity, hyperinsulinemia and insulin resistance, laminitis, dyslipidemia, hyperleptinemia, and altered reproductive cycling [2]. Obesity may play a role in the development of insulin resistance in equine through induction of a pro-inflammatory state [3, 4] and/or elevated plasma lipid concentrations [5]. However, not all obese horses are insulin resistant and, on the contrary, insulin resistance can occur in non-obese animals [2, 6]. Obesity and insulin resistance have been linked with increased risk for laminitis [7, 8]; however, the mechanism by which obesity and insulin resistance/hyperinsulinemia increase susceptibility to laminitis has not yet been clarified. In experimental studies, continuous hyperinsulinemia has been shown to induce laminitis in both ponies and horses [9, 10].

Several studies in equine [3, 11, 12, 13] highlight the role of adipose tissue in the regulation of metabolism and homeostasis through secretion of various cytokines; however the mechanism governing these cytokines in metabolic disorders often remains obscure. Obesity may induce a state of low-grade inflammation by recruiting macrophages into adipose tissue in response to the increased monocyte chemoattractant protein-1 (*MCP-1*) gene expression, and these macrophages may attribute to elevated circulating inflammatory markers in humans and mice such as tumor necrosis factor-alpha (TNF) [14]. Vick *et al*. [3] reported that higher blood *TNF* mRNA expression and plasma TNF concentration were connected with obesity in Thoroughbred mares. However, in non-obese, over-conditioned horses, *MCP-1* or *TNF* gene expression was not different across adipose tissue depots or altered by insulin sensitivity status [12]. In human and rodent obesity, increased mRNA expression of *TNF* is implicated in the induction of insulin resistance through several mechanisms, including inhibition of intracellular signalling from the insulin receptor [15]. Suagee *et al*. [16] reported that acute hyperinsulinemia decreased the transcript abundance of insulin receptor, but had no effect on its protein expression in adipose tissue of Thoroughbred mares.

Obesity also affects the production of adiponectin (ADIPOQ), leptin and retinol binding protein 4 (RBP4). Obesity has been associated with higher plasma concentration of leptin and lower plasma concentration of ADIPOQ in horses [17]. A recent study by Ungru *et al*. [13] reported that elevated circulating ADIPOQ, and decreased circulating leptin and RBP4 were associated with body weight reduction in ponies; however, their expression patterns in tailhead subcutaneous adipose tissue (SAT) did not reflect these changes.

Stearoyl-CoA desaturase (SCD) may play an important role in the pathogenesis of obesity-induced insulin resistance in humans and mice [18]. Mice with *SCD* gene deficiency had increased insulin signalling/sensitivity and were resistant to diet-induced obesity, despite increased food intake [19]. The study by Yao-Borengasser *et al*. [20] showed that the expression of *SCD* is positively correlated with the expression of adiponectin receptor 1 (*ADIPOR1*) in human adipose tissue. To the authors’ knowledge, the role of *SCD* in obesity and/or insulin resistance in equine has not been previously reported.

The present study was carried out to evaluate the effect of grazing on fat deposition, insulin resistance status and SAT gene expressions, and to analyze associations between pasture-associated changes in body condition, insulin resistance and SAT gene expression during the grazing season in Finnhorse mares. The hypotheses were that grazing on cultivated high-yielding pasture (CG) would be accompanied by increased fat deposition, greater basal insulin concentration and alterations in glucose, insulin and non-esterified fatty acids (NEFA) responses to glucose challenge, and more abundant expression of genes potentially associated with insulin resistance in SAT. Additionally, we hypothesized that seasonal change in gene expression would reflect changes in body condition during the grazing season.

## Materials and Methods

### Animals, diets, and experimental design

The experimental procedures were authorized by the National Animal Experiment Board in Finland (Permit Number: ESAVI/2244/04.10.03/2011). Insertion of catheters and skin incisions were performed under local anesthesia with sedation.

Twenty-two mares were grazed either on CG (n = 11) or semi-natural grassland (NG; n = 11) from the end of May to the beginning of September at MTT Agrifood Research Finland in Ypäjä. The coordinates of the pastures are 60.795° N and 23.315° E. The experimental design has been described previously by Särkijärvi *et al*. [21]. In the beginning of the trial, the horses formed equal pairs based on their age, live weight and body condition, medication and reproductive history, and pedigree. The two horses of each pair were then randomly allotted to different pastures. The horses were stable fed according to their needs on maintenance level with similar diets before May samples. The mares had access to CG or NG 24 h a day during the grazing season. Eight mares from each group (in total 16), aged from 6 to 19 yrs. old (mean age 10.46 ± 4.42 SD), were used for intravenous glucose tolerance test (IVGTT) and gene expression profiling as a part of a larger project [21]. One mare from the CG group was excluded from the experiment in July because of laminitis that was not related to treatment; thus, September data presented are derived from 15 mares. The excluded mare had been eating spoiled feed. Spring flood had moved an old hay bale in the paddock and this horse started eating it.

The area of 4.5 ha CG was divided into three equal paddocks (I-III). The grass was dominated with three grass species: tall fescue (*Festuca arundinacea Schreb*.), timothy (*Phleum pratense L*.), and meadow fescue (*Festuca pratensis L*.). All paddocks were fertilized with 82 kg nitrogen per ha at the beginning of the growing season and with 54 kg nitrogen per ha in July. Paddocks II and III were topped in July; old vegetation which was rejected by the horses were cut and collected. There was no need for topping in the paddock I because it was thoroughly eaten by the horses. The suitability of these grass species and their mixtures in the relation to their water soluble carbohydrates content, chemical composition and preferences by Finnhorse mares has been presented previously [22, 23].

Two areas of NG were chosen as trial areas. The first area consisted of 1.2 ha field and 6.5 ha of meadow/forest and the second area of 2.2 ha field and 3.6 ha meadow/forest, respectively. The fields were sown with meadow fescue 7–11 years before the experiment. The fields were fertilized in May with 68 nitrogen per ha as a starter to ensure sufficient grass production. No topping was made during the grazing season. Unpublished research report by Herzon et al. (2013) from semi-natural pastures showed that the horses feed mainly on graminoids and clover. The most preferred species, and those spread widely on the pastures of this study, were meadow foxtail (*Alopecurus pratensis*), tufted hairgrass (*Deschampsia cespitosa*), timothy (*Phleum pratense*), white clover (*Trifolium repens*), as well as dandelion (*Taraxacum officianalis*) and meadowsweet (*Filipendula ulmaria*). Data from 2012–2013 (Herzon et al., 2013, unpublished) showed that the observed feed values of the samples collected during the grazing season (June-September), were adequate to fill the requirements of the grazed horses when the grazing intensity was optimized. Grassland of the type used here may produce 2000–2800 kg dry matter/ha per season, which is less than that of CG (2500–3500 kg DM/ha per season). Previous studies have found that the energy and crude protein values of semi-natural pastures decrease during the season; they are at the highest in the beginning of the summer (leafy phase) and declined when maturing, being lowest in the reproductive phase (Herzon et al., 2013, unpublished).

### Body measurements

Body measurements were determined in May and September for 16 mares used for IVGTT and gene expression. Body weights (BW) were measured with an electric animal scale and body condition score (BCS) evaluated using the Henneke one to nine scoring system [24]. Waist circumference was measured from two-thirds of the distance from the point of the shoulder to the point of the hip as suggested by Carter *et al*. [25]. Subcutaneous fat thickness was measured using an Aloka SSD-500 ultrasound scanner with a 3.5 MHz transducer (Aloka, Tokyo, Japan) at neck and tailhead. The thickness of the neck fat was measured 10 cm down the halfway of the neck length measured from the poll to the withers. Tailhead subcutaneous fat was measured 11 cm cranially and 10 cm laterally from the tailhead.

### Intravenous glucose tolerance test (IVGTT)

On the day prior to IVGTT, horses were restrained in stocks and sedated with detomidine (0.01 mg/kg IV; Domosedan 10 mg/mL, Orion Pharma, Turku, Finland) and butorphanole (0.01 mg/kg IV; Butordol 10 mg/mL, MSD Animal Health, Boxmeer, Netherlands) for the bilateral jugular vein catheter insertion. After having achieved adequate sedation, the hair over the jugular veins was removed and the puncture sites were prepared surgically. Catheters (Mila 14G, Mila International, Kentucky, USA) were then inserted into both jugular veins using a local anesthetic (2% lidocaine, Lidocain 20 mg/mL, Orion Pharma, Turku, Finland) blockade. Finally, the catheters with 3-way valves were heparinized and sutured to skin. In the following morning, after 12 h fasting, mares received a jugular intravenous infusion of 0.3 g of glucose/kg body weight (glucose 300 mg/mL, B. Braun Melsungen AG, Melsungen, Germany). Average infusion times were 5 min 18 sec in May and 5 min 13 sec in September. During the 3-h duration of the IVGTT, venous blood samples were collected at -10, -5, 0, 2, 4, 6, 8, 10, 12, 14, 16, 18, 20, 25, 30, 40, 50, 60, 70, 80, 90, 120, 150 and 180 min relative to the start of glucose infusion. Catheters were flushed with heparin solution (1 mL of heparin/100 mL of 0.9% NaCl) immediately after infusions or blood samplings. Blood samples were collected from the right jugular vein, whereas the infusion of glucose for the IVGTT was administered into the left jugular vein.

The blood samples were immediately placed in EDTA tubes (Vacutainer; BD Medical, Becton Dickinson Oy, Vantaa, Finland) and kept in ice until centrifuged. Blood samples were centrifuged at 2220 × g for 10 min to separate plasma. The plasma was stored in plastic tubes at −20°C until analysis for concentrations of glucose, insulin and NEFA. Plasma NEFA and glucose were determined as described previously by Salin *et al*. [26]. Intra- and inter-assay CVs for glucose measurement were 8.20% and 1.96%, respectively. Intra- and inter-assay CVs for NEFA determination were 7.90% and 1.83%, respectively. Plasma insulin was measured by RIA (Coat-A-Count; Siemens Diagnostics, Los Angeles, CA, USA) according to manufacturer’s instructions. Intra-assay CV for insulin determination was 12.20% and inter-assay CVs were 9.16% and 9.43% for low and medium concentrations, respectively. The detection limit for insulin was 2.56 μIU/mL.

#### Calculations

Plasma glucose, insulin, and NEFA responses to IVGTT were determined as the net incremental area under the response curve (AUC_180_; mmol/L × min for glucose and NEFA; μIU/mL × min for insulin) during the 180 min of IVGTT. The AUC was calculated using the trapezoidal rule [27], where basal concentrations were established as the mean concentration of blood samples taken before glucose infusion (-10 and -5 min for glucose and NEFA and -5 min for insulin). Peaks of glucose and insulin, and the nadir of NEFA concentrations were determined. The clearance rate (CR_180_; %/min) of metabolites during IVGTT was calculated using PROC NLIN of SAS (version 9.2). Exponential curves for the calculation of clearance rate for glucose and NEFA concentrations during IVGTT were fitted using the equations described by Salin *et al*. [26]. Results of the IVGTT for each horse were also analyzed according to the minimal model of glucose and insulin dynamics [28]. Since a large number of implausible solutions for individual IVGTT results was found, minimal model results are not presented for the treatment groups. Instead, they are used only to demonstrate the degree of insulin resistance of horses and its effect on the outcome of minimal model analysis. Based on the estimability of the insulin sensitivity index (Si x 10^-4^/mU/L/min) in minimal model analysis the horses were categorized to two groups. Values 1 < Si < 10 x 10^-4^/mU/L/min were considered normal, whereas very low (Si < 1 x 10^-4^/mU/L/min, n = 10) or very high values (Si > 10 x 10^-4^/mU/L/min, n = 11) were considered problematic to estimate (inestimable).

### Adipose tissue biopsy specimen collection

Neck and tailhead SAT were collected for the gene expression profiling in May and September from the same area where the fat thickness was measured, but on the other side of the horse. Adipose tissue biopsies were performed on the same day of the IVGTT. After the insertion of jugular vein catheters for IVGTT, hair over the sample collection area was removed, and the area was prepared surgically. In addition, local anesthesia (lidocaine 40–100 mg per collection site; Lidocain 20 mg/mL, Orion Pharma, Turku, Finland) was applied to the area prior to skin incision. A 2–3 cm skin incision was made and a 0.5–1.0 g piece of SAT was removed through the incision, snap frozen in liquid nitrogen and stored in -80°C until RNA extraction. Finally, the skin incision was sutured with interrupted pattern.

### RNA extraction and cDNA synthesis

Total RNA was extracted from SAT samples using RNeasy Lipid Tissue Kit (Qiagen GmbH, Hilden, Germany) according to manufacturer’s instructions. The purity of RNA was analyzed by measuring the absorbance at 260 and 280 nm using a NanoDrop ND-1000 spectrophotometer (NanoDrop Technologies, Wilmington, Delaware, USA). RNA quality was assessed using RNA integrity number (RIN) on an Agilent Bioanalyzer 2100 chip electrophoresis system with Agilent RNA 6000 Nano Kit (Agilent Technologies, Santa Clara, CA, USA). Total RNA samples from SAT had 260/280 average absorbance ratio 1.9 and average RIN value 5.8. First-strand cDNA was synthesized with Anchored-Oligo (dT)_18_ primer using Transcriptor First Strand cDNA Synthesis Kit (Roche Diagnostics GmbH, Mannheim, Germany) according to manufacturer’s instructions. The cDNA was then diluted (1:4) with DNase/RNase free water.

### Primer design and quantitative Real-Time RT-PCR

Primers were designed to measure gene expression of the selected genes using the online Primer3 software program [29]. Primer sequences were designed to include at least one exon-exon junction. Primer sequences were searched against NCBI database using BLASTN to determine the uniqueness of selected sequences among annotated sequences in the database. Primer sequences for studied genes and internal control gene are presented in Table 1. The composition of qPCR reactions and the temperature profile of the qPCR were described in Selim *et al*. [30]. The concentration of primers in qPCR was 5 pmol/μl each for forward and reverse primers whereas for MCP-1 the concentration was 20 pmol/μl each for forward and reverse primers. Quantitative real-time PCR was conducted using LightCycler 480 instrument (Roche Diagnostics GmbH, Mannheim, Germany), and each sample was run in quadruplicate. The expression stability was tested for two internal control genes (eukaryotic translation initiation factor 3 subunit K and mitochondrial ribosomal protein L39 (*MRPL39*)) using NormFinder software [31] and *MRPL39* was selected as internal control gene. *MRPL39* has been shown to be one of the most stable internal control genes [32, 33]. The mRNA abundances of *MRPL39* were stable across groups and time points in both SAT. The mRNA abundance was calculated relative to the expression of *MRPL39* as an internal control gene; i.e. the calculation of the expression was based on the difference between the Ct values of the target genes and the internal control gene. The mRNA abundance was presented as delta cycle threshold (ΔCt) values (14-ΔCt). Approximate amplification efficiencies (E) were calculated from the amplification curves as described in Selim *et al*. [30]. Amplification efficiency values for the studied genes and internal control gene are presented in Table 1.

**Table 1:** Studied genes, DNA sequences for forward and reverse primers (5΄-3΄), GenBank accession numbers, the PCR product length and approximate amplification efficiency.

Gene^a^	Sequence 5΄-3΄^b^	GenBank	Length (bp)	E^d^
		accession No.^c^		
***ADIPOQ***	For.GGAGATCCAGGTCTTGTTGG	XM_001499514	162	1.00
	Rev.TCGGGTCTCCAATCCTACAC			
***ADIPOR1***	For.CCCAACCAAAGCTGAAGAAG	XM_001496039	142	1.00
	Rev.ACGTCCCTCCCACACCTTAT			
***ADIPOR2***	For.TTTGTTTGTAAGGTTTGGGAAG	NM_001163830	205	1.00
	Rev.GGCACAGGAAGAACACACAA			
***INSR***	For.TTCCAGCAACTTGATGTGTACC	XM_001496584	174	0.99
	Rev.TCAGCTGCCAGGTTGTTG			
***LEP***	For.CACACGCAGTCAGTCTCCTC	NM_001163980	176	1.00
	Rev.CGGAGGTTCTCCAGGTCAT			
***MCP-1***	For.GGCTCAGCCAGATGCAATTA	NM_001081931	141	0.99
	Rev.ATGGTCTTGAAGTTGGGACACT			
***MRPL39***	For.CCGGCTGGAGATTTATAGCA	XM_001496687	225	0.99
	Rev.CACTCAAATGCATGGCACA			
***RBP4***	For.TGATCTCTCACAACGGTTATTG	NM_001081951	152	0.99
	Rev.GGAGAAGAGAGGGCCAAACT			
***SCD***	For.ACCTACCTCTGGGTGGCTTT	XM_001500364	148	0.99
	Rev.CATTCTGGAAGGCCATCGT			
***TNF***	For.CCCAGAGGGAAGAGCAGTTA	NM_001081819	122	0.99
	Rev.TTGGGGGTTTGCTACAACAT			

^a^ *ADIPOQ*, adiponectin; *ADIPOR1*, adiponectin receptor 1; *ADIPOR2*, adiponectin receptor 2; *INSR*, insulin receptor; *LEP*, leptin; *MCP-1*, monocyte chemoattractant protein-1; *MRPL39*, mitochondrial ribosomal protein L39; *RBP4*, retinol binding protein 4; *SCD*, stearoyl CoA desaturase; *TNF*, tumour necrosis factor-alpha.

^b^For, forward; Rev, reverse; exon-exon junctions are underlined.

^c^GenBank accession numbers are from the databases of the National Center for Biotechnology Information; all sequences were derived from horse (*Equus caballus*).

^d^E, approximate amplification efficiency for each gene calculated from amplification curves as described by Selim et al. [30].

### Statistical analysis

Statistical computations were performed using SAS (SAS Institute Inc., Cary, NC, USA, release 9.3). Residuals of all data at both time points were verified for normality and outliers using the MIXED and UNIVARIATE procedures of SAS. In this analysis, the statistical model included a fixed effect of treatment, and a random effect of pair. Gene expression data were log_2_-transformed to create a normal distribution of data. If data were not normally distributed after log_2_-transformation, data points with highest studentized residuals (>3) were considered outliers. The numbers of observations, which were considered outliers and excluded were two in tailhead (*ADIPOR2*) and three in neck (leptin and *ADIPOR1*) SAT gene expressions.

Final data were analyzed using the MIXED procedure of SAS with a model that included a fixed effect of treatment and a random effect of pair. May values were used as covariates when analyzing September data. Covariate was removed from the model, if the effect was not statistically significant (*P > 0*.*10*). Friedman’s non-parametric test was used to investigate group-related differences in body condition scores. Changes over time were analyzed using a model that included fixed effects of treatment, time, and the interaction between treatment and time, and a random effect of pair. Differences between Si categories were assessed using model that included fixed effect of Si group, using merged IVGTT data. Significance was declared at *P < 0*.*05* and trend at *0*.*05 ≤ P < 0*.*10*. Spearman rank correlation coefficients were calculated for all data using GraphPad Prism 5 software (GraphPad Software, Inc., La Jolla, CA, USA) to identify significant (*P < 0*.*05*) correlations.

## Results

### Body measurements

Results of body measurements have been previously presented in Särkijärvi *et al*. [21] for the 22 horses in the trial. The results of body measurements for the sub-group of 16 horses used in the gene expression profiling and IVGTT were mostly similar to Särkijärvi *et al*. [21] and are presented in Table 2. In May, there were no significant differences between the groups for BW, BCS (median 5.5 vs. 6, interquartile ranges (IQR) were 1 vs. 0.25 for CG and NG, respectively; *P = 0*.*35*) or waist circumference (*P = 0*.*65*). In September, the CG group had a higher BW (*P = 0*.*02*) and larger waist circumference (*P = 0*.*03*) compared to NG. The CG group had higher (*P = 0*.*05*) median BCS (7, IQR = 0.25) than the NG mares (5.4, IQR = 0.43) after the grazing season. There was no difference between the groups in neck fat thickness before or after grazing (*P>0*.*10*). However, CG mares had a smaller tailhead fat thickness compared to NG mares in May (*P = 0*.*04*), but this difference disappeared in September. Higher waist circumference and fat thickness of neck and tailhead were observed for all mares in September compared to May (*P<0*.*05*).

**Table 2:** Effect of pasture type on body measurements of Finnhorse mares.

	Time relative to the grazing season^b^								
	Before grazing (May)				After grazing (September)				
Parameter^a^	NG	CG	SEM	P-value	NG	CG	SEM	*P-value*	Covariate^c^
**BW, kg**	545	552	12.35	0.71	572	618	10.21	0.02	0.01
**SFT at neck, cm**	0.9	1.0	0.09	0.51	1.1	1.2	0.07	0.17	0.35
**SFT at tailhead, cm**	2.3	1.9	0.16	0.04	2.7	3.4	0.36	0.24	0.09
**Waist circumference, cm**	208	210	2.99	0.65	212	219	2.23	0.03	0.85

^a^SFT, subcutaneous fat thickness.

^b^Finnhorse mares were grazing either on cultivated high-yielding pasture (CG) or semi-natural grassland (NG) from the end of May to the beginning of September.

^c^Covariate (May) was removed from the model, if the effect was not statistically significant (*P > 0*.*10*).

### Response to IVGTT

**Fig 1:**
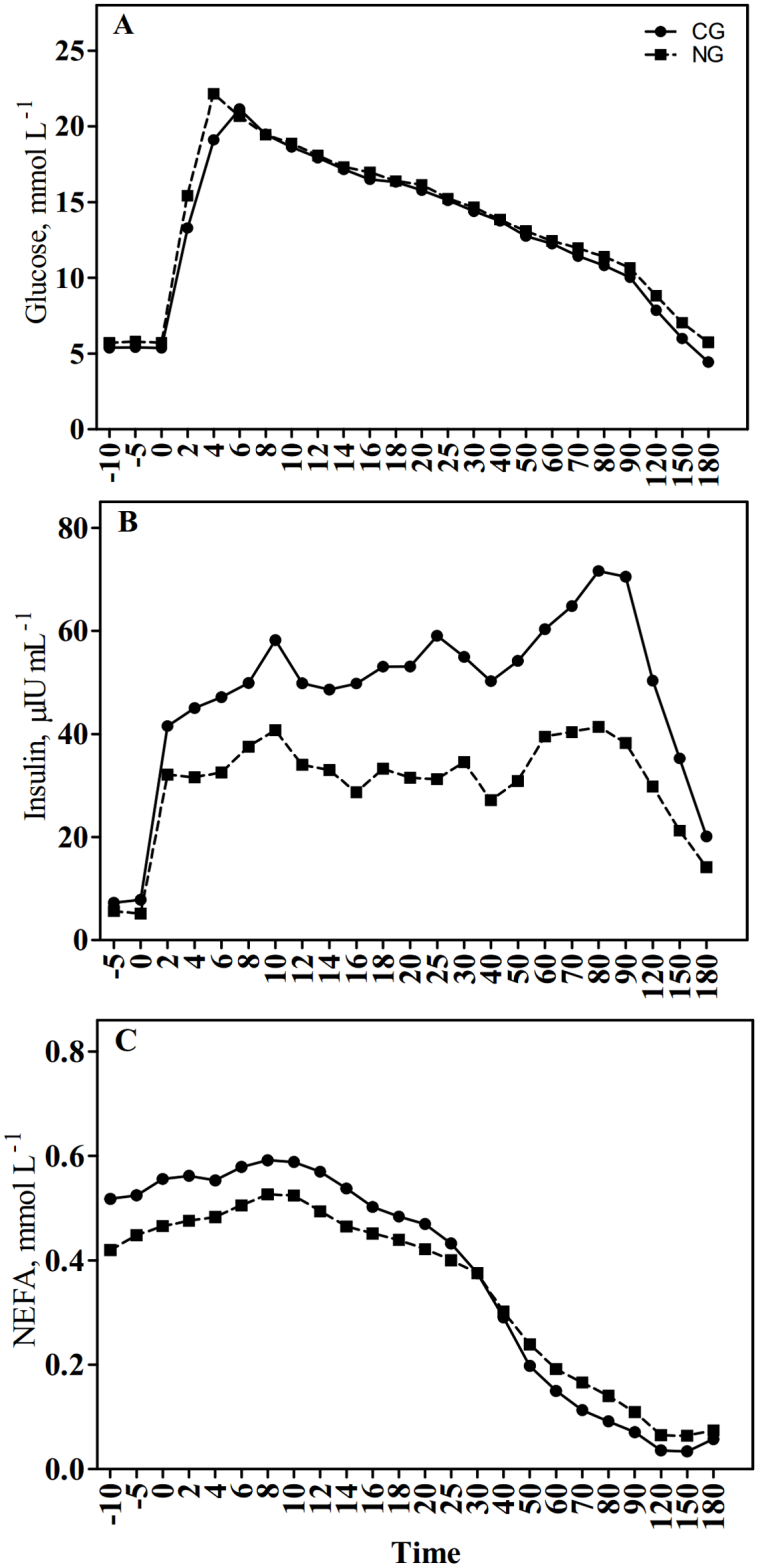
Effect of grazing on cultivated high-yielding pasture (CG) or semi-natural grassland (NG) on plasma glucose (A), insulin (B), and (C) NEFA concentrations after i.v. glucose tolerance test (IVGTT; 0.3 g of glucose/kg of BW).

**Fig 2:**
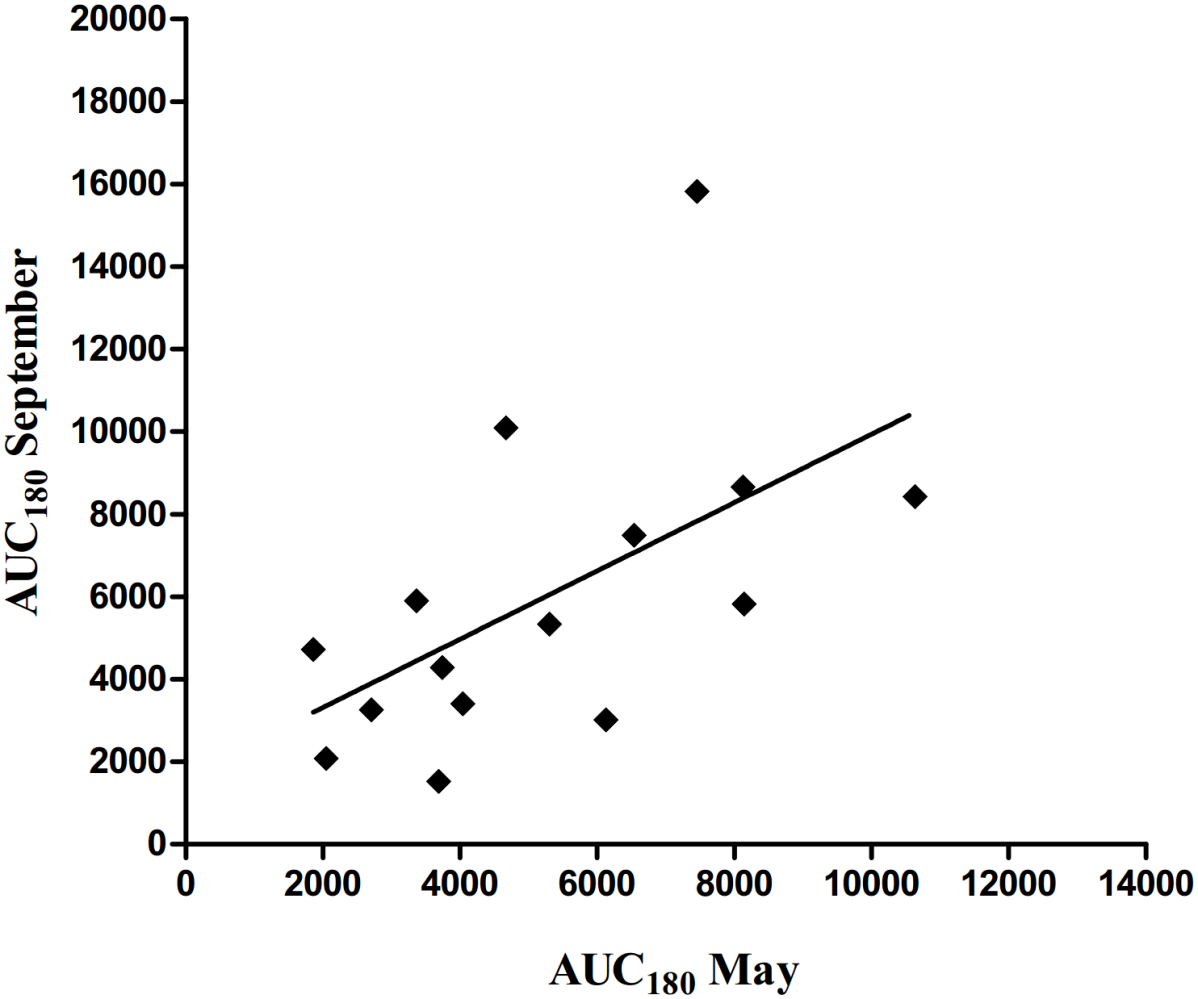
Correlation (r = 0.60, *P = 0*.*02*) between insulin area under curve during the 180 min of i.v. glucose tolerance test (IVGTT; 0.3 g of glucose/kg of BW) (AUC_180_) before and after the grazing season.

Plasma glucose, insulin and NEFA responses during IVGTT are given in Table 3 and Fig 1. Before the grazing season (May), no significant differences were observed between CG and NG except for basal glucose concentration which was lower in CG than in NG (*P = 0*.*01*). This was also observed after the grazing season (*P = 0*.*04*). The CR_180_ of glucose was greater (*P = 0*.*03*) in CG mares compared to NG mares at the end of the grazing season. Glucose peak and AUC_180_ were not affected by treatments in September. The CG mares tended to have higher basal and peak insulin concentration (*P = 0*.*07* and *P = 0*.*08*, respectively) in September than the NG mares, while insulin AUC_180_ was not affected. The treatments did not influence basal and nadir NEFA concentrations at the end of the grazing season. However, the absolute values of NEFA AUC_180_ (*P = 0*.*03*) and NEFA CR_180_ (*P = 0*.*02*) were greater in CG than in NG. Positive correlation (r = 0.60, *P = 0*.*02*) between insulin AUC_180_ in May and September was found (Fig 2). Additionally, we observed differences in insulin response to IVGTT between individuals within each group in May and September (S1 Fig). It was remarkable that three individuals (2 CG mares in May and 1 NG mare in September) did not develop hyperinsulinemia during IVGTT (reference range less than 20 μIU/ml).

**Table 3:** Plasma glucose, insulin and NEFA response to i.v. glucose tolerance test (IVGTT, 0.3 g of glucose i.v. /kg of BW) of Finnhorse mares before and after the grazing season.

	Time relative to the grazing season^b^								
	Before grazing (May)				After grazing (September)				
Item^a^	NG	CG	SEM	*P-value*	NG	CG	SEM	*P-value*	Covariate^c^
**Glucose**									
** Basal (mmol/L)**	5.8	5.3	0.08	0.01	5.8	5.4	0.14	0.04	NS
** Peak (mmol/L)**	22.2	22.2	0.78	0.97	22.6	21.7	0.45	0.19	0.07
** CR**_**180**_ **(%/min)**	0.79	0.81	0.06	0.72	0.72	0.81	0.04	0.03	NS
** AUC**_**180**_ **(mmol/L x min)**	895.3	878.0	44.35	0.79	927.0	902.8	50.21	0.70	NS
**Insulin**									
** Basal (μIU/mL)**	5.1	4.9	1.02	0.88	5.5	7.3	0.59	0.07	0.01
** Peak (μIU/mL)**	48.0	54.9	8.77	0.60	47.1	76.9	9.89	0.08	0.05
** AUC**_**180**_ **(μIU/mL x min)**	4,869	6,431	1,052	0.33	4,698	7,465	1,107	0.13	0.06
**NEFA**									
** Basal (mmol/L)**	0.41	0.41	0.05	0.98	0.43	0.52	0.04	0.17	NS
** Nadir (mmol/L)**	0.05	0.03	0.01	0.26	0.05	0.04	0.01	0.33	0.02
** CR**_**180**_ **(%/min)**	2.06	2.07	0.18	0.96	1.64	2.24	0.15	0.02	NS
** AUC**_**180**_ **(mmol/L x min)**	-49.6	-49.7	4.74	0.98	-43.3	-63.5	4.52	0.03	0.06

^a^Basal, average concentration at 10 and 5 min before IVGTT; CR_180_, clearance rate during first 180 min of IVGTT; AUC_180_, area under the curve during the first 180 min of IVGTT; NEFA, non-esterified fatty acids.

^b^NG, semi-natural grassland group; CG, cultivated high-yielding pasture group.

^c^Covariate (May) was removed from the model, if the effect was not statistically significant (*P > 0*.*10*).

Results of minimal model analysis based on Si classification are shown in Table 4. Si estimates were classified as normal in 10 tests out of total number of 31 individual IVGTT, and in 21 tests Si estimates suggested severe insulin resistance or showed problems in estimability. In the tests categorized as normal, horses tended to have higher basal insulin concentration (*P = 0*.*07*), had higher peak insulin concentration (*P = 0*.*04*) and greater insulin AUC during the first 60 min of IVGTT (*P = 0*.*04*) than in the other group. No differences in glucose and NEFA responses were observed.

**Table 4:** Plasma glucose and insulin response during i.v. glucose tolerance test (IVGTT, 0.3 g of glucose i.v. /kg of BW) in different categories of insulin sensitivity classification.

	Insulin sensitivity status^b^			
	1≤Si<10	Si<1 or Si≥10		
Item^a^	Normal	Resistant or inestimable	SEM	*P-value*
**N**	10	21		
**Glucose**				
** Basal (mmol/L)**	5.7	5.5	0.14	0.34
** Peak (mmol/L)**	22.8	21.9	0.56	0.24
** AUC**_**60**_ **(mmol/l x min)**	536.1	555.2	15.20	0.31
**Insulin**				
** Basal (μIU/mL)**	6.9	5.1	0.81	0.07
** Peak (μIU/mL)**	71.7	48.6	8.70	0.04
** AUC**_**60**_ **(μIU/mL x min)**	2646	1738	346.1	0.04
**NEFA**				
** Basal (mmol/L)**	0.42	0.45	0.045	0.51
** Decrease (mmol/L)**	0.37	0.42	0.039	0.31

^a^Basal, average concentration at 10 and 5 min before IVGTT; AUC_180_, area under the curve during the first 180 min of IVGTT; NEFA, non-esterified fatty acids.

^b^Si, the insulin sensitivity index (× 10^-4^ min^-1^/μIU/mL).

### Gene expression in neck subcutaneous adipose tissue

There were no differences in the gene expression of *ADIPOQ*, *ADIPOR1/2*, insulin receptor, leptin, *MCP-1*, *RBP4*, *SCD* or *TNF* between the groups before or after the grazing season (Table 5). The mRNA abundances coding for insulin receptor (*P = 0*.*04*), *RBP4* (*P = 0*.*06*), leptin (*P = 0*.*004*) and *MCP-1* (*P = 0*.*04*) were down-regulated in September compared to May across groups.

**Table 5:** Relative mRNA abundance of neck subcutaneous adipose tissue genes [Log_2_ (14-ΔCt)] of Finnhorse mares grazed either on cultivated high-yielding pasture or semi-natural grassland.

	Time relative to the grazing season^b^								
	Before grazing (May)				After grazing (September)				
Gene^a^	NG	CG	SEM	*P-value*	NG	CG	SEM	*P-value*	Covariate^c^
***ADIPOQ***	4.09	4.16	0.07	0.52	4.05	4.03	0.13	0.91	0.78
***ADIPOR1***	3.67	3.74	0.06	0.44	3.70	3.87	0.07	0.14	0.12
***ADIPOR2***	3.58	3.71	0.07	0.22	3.61	3.72	0.15	0.49	0.85
***INSR***	4.33	4.25	0.08	0.39	4.20	4.02	0.09	0.23	0.98
***LEP***	4.10	4.05	0.03	0.23	3.97	3.77	0.09	0.14	0.43
***MCP-1***	3.76	3.71	0.05	0.20	3.65	3.51	0.10	0.35	0.32
***RBP4***	3.26	3.51	0.14	0.17	3.02	2.87	0.35	0.75	0.73
***SCD***	3.75	3.81	0.11	0.71	3.70	4.02	0.19	0.25	0.57
***TNF***	2.41	2.90	0.21	0.15	2.24	2.31	0.29	0.67	0.70

^a^ *ADIPOQ*, adiponectin; *ADIPOR1*, adiponectin receptor 1; *ADIPOR2*, adiponectin receptor 2; *INSR*, insulin receptor; *LEP*, leptin; *MCP-1*, monocyte chemoattractant protein-1; *RBP4*, retinol binding protein 4; *SCD*, stearoyl-CoA desaturase; *TNF*, tumor necrosis factor-alpha.

^b^NG, semi-natural grassland group; CG, cultivated high-yielding pasture group.

^c^Covariate (May) was removed from the model, if the effect was not statistically significant (*P > 0*.*10*).

### Gene expression in tailhead subcutaneous adipose tissue

In May, CG mares had tendencies for lower mRNA abundance of *ADIPOQ* (*P = 0*.*08*), and *ADIPOR2* (*P = 0*.*08*), and significantly lower mRNA expression of *SCD* (*P = 0*.*02*) compared to NG, but these differences disappeared in September (Table 6). In addition, up-regulation of *MCP-1* mRNA was observed in CG compared to NG before grazing (*P = 0*.*02*), but there was no difference in *MCP-1* mRNA abundance between the groups after grazing (Table 6). Gene expressions of *ADIPOR1*, insulin receptor, leptin, *RBP4* or *TNF* were not different between the groups (Table 6). We observed down-regulation of insulin receptor (*P<0*.*001*), *MCP-1* (*P<0*.*001*) and *RBP4* (*P = 0*.*03*) in September compared to May in both groups. Up-regulation of *ADIPOQ* (*P = 0*.*03*), *ADIPOR1* (*P = 0*.*03*) and *ADIPOR2* (*P = 0*.*06*) was found in September compared to May across the groups.

**Table 6:** Relative mRNA abundance of tailhead subcutaneous adipose tissue genes [Log_2_ (14-ΔCt)] of Finnhorse mares grazed either on cultivated high-yielding pasture or semi-natural grassland.

	Time relative to the grazing season^b^								
	Before grazing (May)				After grazing (September)				
Gene^a^	NG	CG	SEM	*P-value*	NG	CG	SEM	*P-value*	Covariate^c^
***ADIPOQ***	4.20	4.11	0.03	0.08	4.34	4.28	0.09	0.69	0.56
***ADIPOR1***	3.70	3.72	0.05	0.79	3.92	3.89	0.11	0.90	0.59
***ADIPOR2***	3.79	3.67	0.04	0.08	3.88	3.90	0.11	0.89	0.24
***INSR***	4.29	4.33	0.07	0.69	4.04	3.90	0.07	0.18	0.67
***LEP***	4.00	3.99	0.04	0.87	3.87	3.94	0.06	0.45	0.96
***MCP-1***	3.63	3.91	0.07	0.02	3.50	3.27	0.10	0.10	0.07
***RBP4***	3.11	3.01	0.16	0.66	2.80	2.50	0.19	0.24	0.12
***SCD***	4.02	3.87	0.05	0.02	4.12	4.20	0.13	0.63	0.71
***TNF***	2.24	2.42	0.19	0.54	1.94	1.84	0.32	0.83	0.96

^a^ *ADIPOQ*, adiponectin; *ADIPOR1*, adiponectin receptor 1; *ADIPOR2*, adiponectin receptor 2; *INSR*, insulin receptor; *LEP*, leptin; *MCP*-*1*, monocyte chemoattractant protein-1; *RBP4*, retinol binding protein 4; *SCD*, stearoyl-CoA desaturase; *TNF*, tumor necrosis factor-alpha.

^b^NG, semi-natural grassland group; CG, cultivated high-yielding pasture group.

^c^Covariate (May) was removed from the model, if the effect was not statistically significant (*P > 0*.*10*).

### Correlations between gene expressions and body measurements

**Fig 3:**
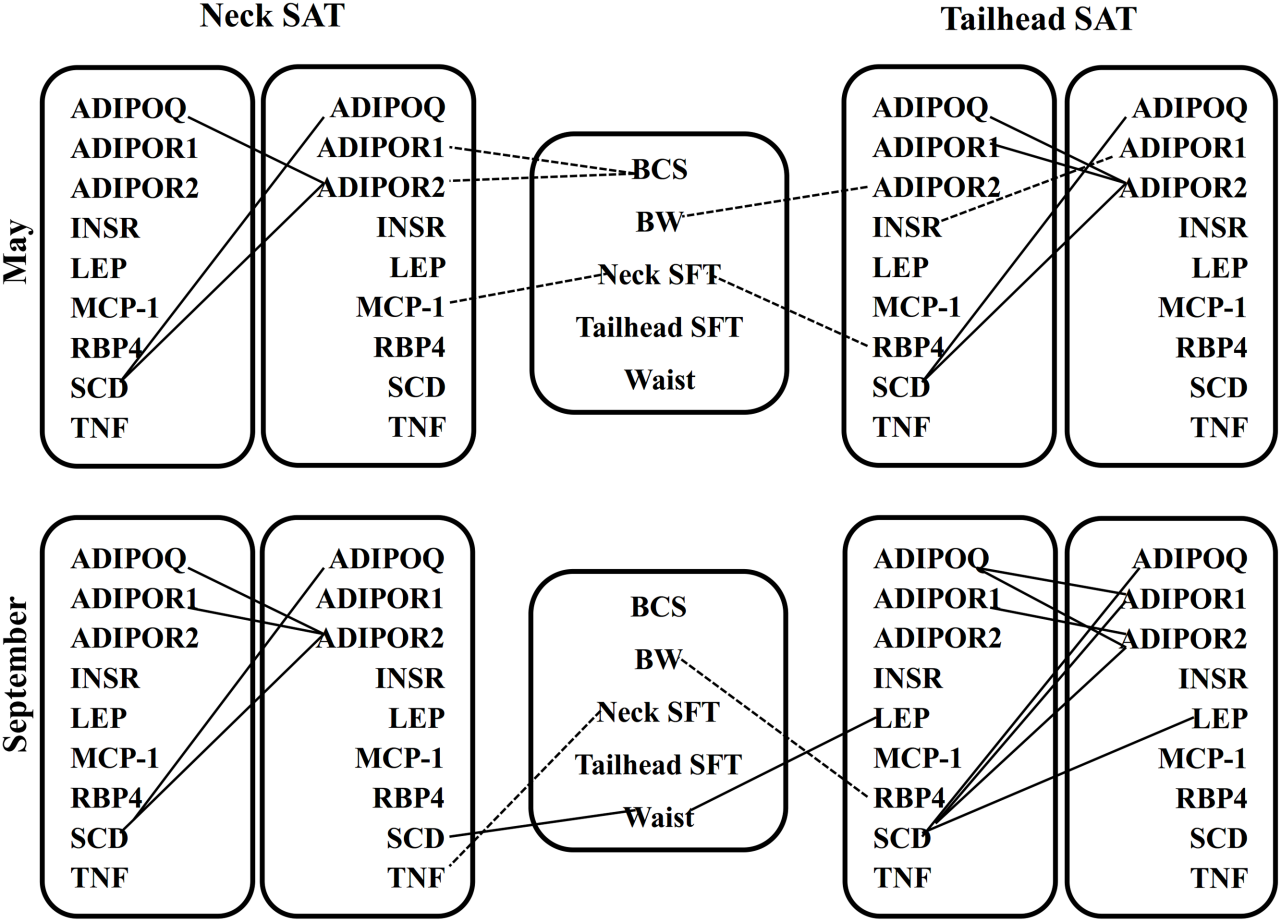
Relationships between body measurements and gene expression measurements in subcutaneous neck and tailhead adipose tissue in May and September as revealed by Spearman’s correlation coefficients. The correlations shown had an *R>0*.*50* and *P<0*.*05*. Solid lines represent positive and dashed lines represent negative correlations. SFT, subcutaneous fat thickness; *ADIPOQ*, adiponectin; *ADIPOR1*, adiponectin receptor 1; *ADIPOR2*, adiponectin receptor 2; *INSR*, insulin receptor; *LEP*, leptin; *MCP*-*1*, monocyte chemoattractant protein-1; *RBP4*, retinol binding protein 4; *SCD*, stearoyl Co-A desaturase; *TNF*, tumor necrosis factor-alpha.

The correlations between gene expression and body measurements are presented in Fig 3. The correlations shown had an R-value of >0.50. In May, BCS was negatively correlated with neck *ADIPOR1* and *ADIPOR2* mRNA abundance. There was a significant negative relationship between neck *MCP-1* expression and neck fat thickness. Body weight showed negative correlation with tailhead *ADIPOR2* gene expression. Tailhead *RBP4* mRNA abundance had negative correlation with neck fat thickness. In September, waist circumference showed positive correlations with tailhead leptin and neck *SCD* mRNA expressions. A significant negative correlation was observed between tailhead *RBP4* expression and BW. Neck *TNF* mRNA had a negative correlation with neck fat thickness.

### Correlations between gene expressions in subcutaneous adipose tissue

The correlations between gene expression in SAT are presented in Fig 3 and had an R-value of >0.50. In May, *ADIPOQ* had a strong positive correlation with *ADIPOR2* mRNA abundance in both SAT. Tailhead *ADIPOR1* and *ADIPOR2* gene expressions were positively correlated with each other. Tailhead insulin receptor gene expression showed negative correlation with tailhead *ADIPOR1* mRNA expression. The mRNA abundance of *SCD* showed a significant positive correlation with *ADIPOQ* and *ADIPOR2* in both SAT. In September, a strong positive correlation was observed between tailhead *ADIPOQ* and *ADIPOR1* and *ADIPOR2* gene expression. However, neck *ADIPOQ* was positively correlated with neck *ADIPOR2* only. Positive correlations were observed between *ADIPOR1* and *ADIPOR2* mRNA expressions in both SAT. Tailhead *SCD* mRNA abundance was positively correlated with tailhead *ADIPOQ*, *ADIPOR1*, *ADIPOR2* and leptin gene expression. However, in neck SAT, *SCD* was positively correlated with *ADIPOQ* and *ADIPOR2* mRNA abundance.

## Discussion

In this study, horses were grazing 98 days on two types of pastures. Horses on CG had increased BW, BCS, and waist circumference compared to horses grazing on NG. Previous studies [3, 5], utilizing the 9-point BCS scale [24], have described horses with BCS ≥ 7 as obese. In the current study, median BCS of the CG group was 7 compared to 5.4 in the NG group after the grazing season, suggesting that mares in the CG group were overweight. In addition, the large increase in waist circumference in the CG group in September may indicate that the CG mares might have accumulated more visceral fat than subcutaneous fat during the grazing season.

### Status of insulin resistance

The pasture type had only moderate effects on responses during IVGTT. Horses on CG tended to have higher basal and peak insulin concentrations in September than horses on NG. Increased glucose clearance rate of CG in September indicates that higher BW and BCS gain did not compromise the ability of these horses to control plasma glucose. However, greater basal and peak insulin concentrations might indicate a compensatory insulin secretion in response to decreased insulin sensitivity or responsiveness of peripheral tissues in CG compared to NG. Earlier studies have shown that increased consumption of feeds with high content of non-structural carbohydrates is associated with insulin resistance in the context of obesity [34, 35]. However, Suagee *et al*. [36] showed recently that high glycaemic diet did not alter peripheral insulin sensitivity in horses that had BCS < 7.5. Further, Bailey et al. [37] observed that pasture feeding with grass rich in fructans did not increase insulin resistance of non-obese healthy ponies, whereas ponies previously predisposed to laminitis developed increased insulin resistance. In the present study, a positive correlation was found between insulin AUC’s before and after the grazing season. This finding together with only moderate effect of pasture type on insulin AUC suggest that in non-obese horses, previous health status or nutritional history may be more important factors affecting insulin function than pasture feeding.

Greater decrease in plasma NEFA during IVGTT in the CG mares, and the lack of difference in basal NEFA concentration, suggest that increased energy intake did not compromise insulin sensitivity/responsiveness of adipose tissue. In obese horses insulin resistance reduces the ability of insulin to inhibit hormone-sensitive lipase, resulting in elevated plasma NEFA concentrations [5]. However, Carter *et al*. [35] observed that feeding horses for weight-gain over 16 weeks had no effect on plasma NEFA concentrations despite induced obesity, indicating that longer period of insulin resistance may be needed for the development of major changes in lipid metabolism. Suagee *et al*. [36] observed that meal-induced NEFA suppression was reduced in non-obese horses after only 90 d feeding of high glycemic diet, showing that insulin resistance in adipose tissue takes time to develop. In the present study, the differences between groups in energy and carbohydrate intake were probably too moderate to induce major effects on adipose tissue insulin sensitivity, or grazing season was too short to affect lipid metabolism of the horses.

An alternative or complementary explanation for the lack of effects of more abundant energy supply from CG could be the initial insulin resistance status of the horses (e.g. horses might have been relatively insulin resistant before the grazing season). Several horses showed blunted insulin response during IVGTT (S1 Fig), suggesting impaired pancreatic β cell response to glucose [38]. The individual differences in insulin secretion in response to glucose stimulus were largely retained during the grazing period, as indicated by the positive correlation of insulin AUC during IVGTT in May and September. Furthermore, based on the Si classification results, a large proportion of horses was either insulin resistant or had implausible Si estimates, and these horses had lower insulin response during IVGTT (Table 4).

Previously minimal model analysis of frequently sampled IVGTT has been successfully used in horses [38–39]. Treiber et al. [40] reported that the lowest quintile of Si estimates was 0.14–0.78 x 10^-4^/mU/L/min in healthy Thoroughbred horses, whereas highest quintile was 3.05–5.94 x 10^-4^/mU/L/min. On the other hand, the study by Bamford et al. [41] demonstrated breed-related differences in the insulin responses of horses and ponies to intravenous glucose test, showing that Andalusians were relatively insulin resistant, having mean Si of 0.99 x 10^-4^/mU/L/min, whereas in Standardbred horses mean Si was 5.43 x 10^-4^/mU/L/min. Based on these previous studies, classifying Si below 1 as insulin resistant seems justified, whereas Si over 10 x 10^-4^/mU/L/min would represent extreme insulin sensitivity. It is likely that these extreme values of Si were also contributed by the limited pancreatic capacity to secrete insulin in response to glucose stimulation. However, based on the current approach, IVGTT without exogenous insulin, it is not possible to judge between the above-mentioned alternatives. Additionally, relatively long duration of glucose infusion may have contributed the problems in Si estimation. Nevertheless, the IVGTT was not originally designed and optimized for minimal model analysis, and the problems in estimation are probably due to the fact that exogenous insulin was not injected during the test [39]. The current results emphasize the importance of applying exogenous insulin during IVGTT, if minimal model is used in the analysis of glucose-insulin dynamics in horses with a relatively high degree of insulin resistance.

### Gene expression in adipose tissue

Adipose tissue constitutes a dynamic endocrine organ, secreting hormones and inflammatory mediators which have been involved in the inflammatory response and insulin resistance in dogs, cats, and horses [42]. Adiponectin has been shown to modulate fatty acid metabolism and energy homeostasis [43]. Previous studies in horses by Kearns *et al*. [17] and Gordon *et al*. [44] showed that circulating ADIPOQ was negatively correlated with fat mass, percent body fat and BCS. In the current study, negative correlations between neck *ADIPOQ* receptors and BCS were detected in May but not in September. Tsuchida *et al*. [45] reported that in mice, the expression of *ADIPOR1*/2 was correlated with ADIPOQ sensitivity. Therefore, the observed positive correlations between *ADIPOQ* and its receptors in the current study suggest a synchronous regulation of *ADIPOQ* and its receptors at transcriptional level. In addition, up-regulation of *ADIPOQ* and its receptors in tailhead SAT but not in neck SAT in September compared to May was observed across treatments. Fu *et al*. [46] found that *ADIPOQ* expression promotes proliferation and differentiation of adipocytes, and enhances insulin sensitivity and lipid accumulation in mature adipocytes. A recent study in horses by Bruynsteen *et al*. [33] suggested that SAT (loin and tailhead) is more stimulated to differentiate pre-adipocytes into adipocytes than other adipose tissue depots. Our results may indicate that insulin sensitivity, and differentiation and accumulation of adipocytes were more enhanced in tailhead SAT than in neck SAT.

We detected down-regulation in the mRNA abundance of insulin receptor in September compared to May in both groups. Increased basal insulin concentrations during the grazing season (*P = 0*.*16*), reduced insulin receptor gene expression in SAT which could possibly indicate a negative feedback mechanism of insulin on the insulin signaling pathway in SAT [16]. Basal blood samples were collected after fasting which may mask the potential time effect on insulin concentration. Suagee *et al*. [16] found that a six-h insulin infusion to insulin sensitive horses decreased insulin receptor transcript abundance in adipose tissue but did not affect its protein expression. A recent study by de Laat et al. [47] reported a reduction in insulin receptor gene expression in lamellar tissue of horses during the development (24 h) and acute (46 h) stages of insulin-induced laminitis compared to control horses. In the current study, the observed higher insulin concentration in CG was not large enough to induce significant effect on the mRNA expression of insulin receptor.

Gene expression of *SCD* in both neck and tailhead SAT was not different between treatments in September. However, time effect was observed in tailhead SAT characterized by a higher mRNA abundance in September compared to May. Stearoyl-CoA desaturase is the rate-limiting enzyme for fat accumulation in adipose tissue, converting saturated fatty acids into monounsaturated fatty acids [48]. The expression of *SCD* was increased in tailhead but not in neck SAT during the grazing season. This was in line with greater fat thickness in tailhead compared to neck, and thus lipogenesis may differ among fat depots. The strong positive correlation between *SCD* and *ADIPOQ* and its receptors suggests that *ADIPOQ* and *SCD* in horses, as well as in humans [20], may be closely linked through a selective mechanism in adipose tissue regarding lipogenesis and potentiation of *ADIPOQ* signalling. However, the link between the regulation of SCD and insulin sensitivity in equine is unclear.

In the current study, tailhead *RBP4* mRNA showed negative correlation with BW of horses in September. Ungru *et al*. [13] observed a significant reduction in serum RBP4 with the body weight reduction program, but there were no differences in the mRNA expression of *RBP4* in tailhead SAT between insulin sensitive and insulin resistant ponies. Ungru *et al*. [13] stated that serum RBP4 was closely linked to adiposity probably independently of insulin resistance.

A significant down-regulation of *MCP*-*1* in both neck and tailhead SAT during the grazing season suggests that the regulation of *MCP-1* in SAT of horses may not be tightly linked to the state of fat reserves. Alternatively, the significant effect of fattening in the mRNA expression of *MCP-1* may not be observed until the onset of obesity or following a prolonged period of obesity. Our results were in line with Burns *et al*. [12] who observed that the *MCP-1* mRNA abundance was not related to insulin sensitivity status, and *MCP-1* expression was not different across adipose tissue depots in non-obese or over-conditioned horses.

We observed down-regulation of leptin mRNA in neck SAT across the groups in September compared to May. Fitzgerald and McManus [49] reported an increase in the average blood concentration of leptin in young and mature Thoroughbred mares from July to September. Ungru *et al*. [13] observed a significant reduction in serum leptin after body weight reduction in obese insulin resistant ponies. Gentry *et al*. [50] reported that other factors than body fat mass determine the relative levels of leptin production and secretion (low vs. high) in light horse mares under conditions of BCS between 6.5 and 8. The seasonal rise of circulating leptin in late summer and autumn compared to winter may not be entirely dependent on body fat mass [49, 50], and other factors such as environmental influences and seasonal reproductive activity may be involved. In the current study, the down-regulation of neck leptin, with no difference in tailhead leptin gene expression, during the grazing season suggests that seasonal changes of leptin gene expression in SAT of Finnhorse mares may be regulated by other factors than body fat mass. In line with this, we did not find any correlation between leptin mRNA abundance and BW, BCS or subcutaneous fat thickness.

Frank *et al*. [51] and McFarlane *et al*. [52] have shown that the concentrations of ACTH and alpha-melanocyte stimulating hormone (α-MSH) in plasma are highest in the autumn (August-October) in horses. This adaptation helps the animals to prepare themselves for the metabolic and nutritional demands during the winter [53]. Alpha-melanocyte stimulating hormone plays a role in energy metabolism and has anti-inflammatory effects that involve decreasing the production of cytokines and other factors contributing to inflammation [54]. Based on these previous studies, we suggest that the observed down-regulation of leptin, *RBP4* and *MCP-1* gene expression in September compared to May might reflect a seasonal adaptation of horses to the winter season.

We did not detect any treatment- or time-related differences in the mRNA abundance of *TNF*. Our results were in line with Burns *et al*. [12] who observed that *TNF* mRNA expression was not different between insulin resistant and insulin sensitive horses across adipose tissue depots. Similarly, Carter [11] reported that obesity induced insulin resistance was not accompanied by changes in *TNF* mRNA expression in neck SAT. In contrast, Vick *et al*. [3] showed that obesity and insulin resistance were associated with elevated plasma TNF concentrations in horses and Treiber *et al*. [55] found that ponies affected with equine metabolic syndrome had higher plasma TNF concentrations. In addition, Saleri *et al*. [56] postulated that TNF may strongly modulate glucose and lipid metabolism in mares with higher adiposity during the lactation period. Tumor necrosis factor-alpha has been shown to be over-expressed in white adipose tissue in various animal models of obesity. However, the mechanisms of insulin resistance are not completely understood and there are contradictory results [3, 11, 12, 55], whether obesity is or is not related to low-grade inflammation in equine. Our results suggest that moderate increase of fatness during the grazing season may not be associated with low-grade inflammation of SAT in horses.

Giles *et al*. [1] and Johnson *et al*. [57] have shown that certain native breeds are more at risk of becoming obese. This can be explained by adaptation to the seasonality of ancestral environments of native breeds. Obese body condition at the end of summer can be viewed as a natural survival strategy for poorer feed quality and inescapable weight loss during winter months. Our hypothesis was that Finnhorse is a high-risk breed that develop obesity and obesity-associated metabolic diseases if horses have unrestricted access to cultivated high-yielding pasture. However, based on our results, we suggest that Finnhorses became overweight or moderately obese at the end of summer, but they stayed healthy without signs of obesity-associated metabolic diseases.

## Conclusions

We conclude that fattening associated with grazing on cultivated high-yielding pasture induced moderate changes in glucose, insulin and NEFA responses during IVGTT but did not compromise the ability of the horses to control plasma glucose concentrations and adipose tissue lipolysis. Significant temporal differences in the gene expression profiles during the grazing season were observed. Based on the subcutaneous neck and tailhead adipose tissue gene expressions, the BCS and weight gain during the grazing season were not associated with increased insulin resistance. These findings support the hypothesis that obesity and severe hyperinsulinemia are required to produce alterations in insulin resistance status in equine. Finally, our results indicate that grazing on cultivated high-yielding pasture does not increase the risk for metabolic diseases in Finnhorse mares that have normal BCS at the beginning of the grazing season.

## Supporting Information

S1 Fig Individual’s insulin response (μIU/mL) to i.v. glucose tolerance test (IVGTT; 0.3 g of glucose/kg of BW) before and after the grazing season. NG, semi-natural grassland group; CG, cultivated high-yielding pasture group.
(TIF)
