# Supplementary figures and images for: Relationships among Body Condition, Insulin Resistance and Subcutaneous Adipose Tissue Gene Expression during the Grazing Season in Mares
Source: PLoS One. 2015 May 4;10(5):e0125968. doi: 10.1371/journal.pone.0125968 (PMC4418745; doi:10.1371/journal.pone.0125968)

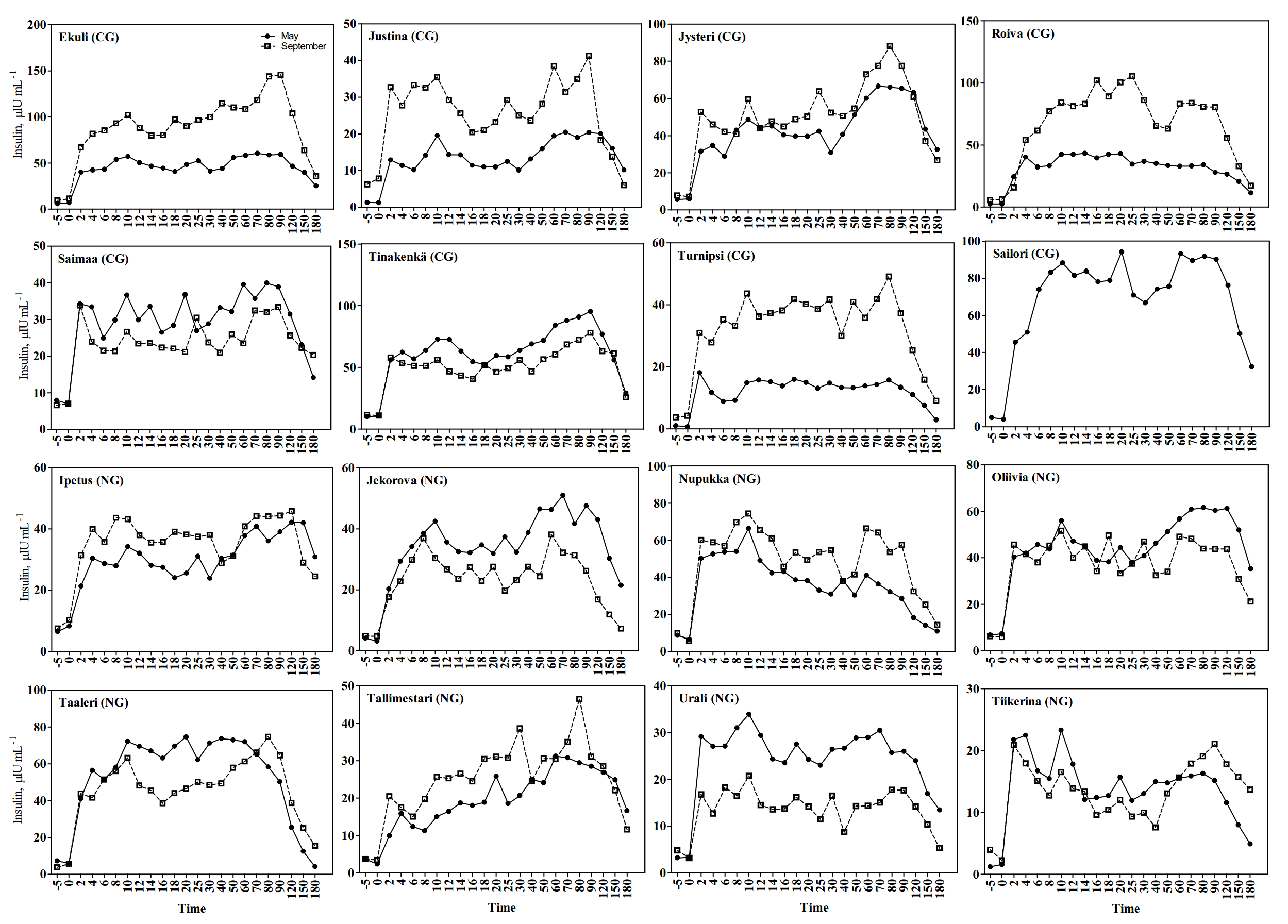

Supplement: S1 Fig — NG, semi-natural grassland group; CG, cultivated high-yielding pasture group. (TIF) [file pone.0125968.s001.tif]
